# Safe using messages may not be enough to promote behaviour change amongst injecting drug users who are ambivalent or indifferent towards death

**DOI:** 10.1186/1477-7517-6-18

**Published:** 2009-07-25

**Authors:** Peter G Miller

**Affiliations:** 1 School of Psychology, Faculty of Health, Medicine, Nursing and Behavioural Sciences, Deakin University, Waterfront campus, Level 3, 27 Brougham Street, Geelong, Victoria 3217, Australia

## Abstract

**Background:**

Health promotion strategies ultimately rely on people perceiving the consequences of their behaviour as negative. If someone is indifferent towards death, it would logically follow that health promotion messages such as safe using messages would have little resonance. This study aimed to investigate attitudes towards death in a group of injecting drug users (IDUs) and how such attitudes may impact upon the efficacy/relevance of 'safe using' (health promotion) messages.

**Methods:**

Qualitative, semi-structured interviews in Geelong, Australia with 60 regular heroin users recruited primarily from needle and syringe programs.

**Results:**

Over half of the interviewees reported having previously overdosed and 35% reported not engaging in any overdose prevention practices. 13% had never been tested for either HIV or hepatitis C. Just under half reported needle sharing of some description and almost all (97%) reported previously sharing other injecting equipment. Many interviewees reported being indifferent towards death. Common themes included; indifference towards life, death as an occupational hazard of drug use and death as a welcome relief.

**Conclusion:**

Most of the interviewees in this study were indifferent towards heroin-related death. Whilst interviewees were well aware of the possible consequences of their actions, these consequences were not seen as important as achieving their desired state of mind. Safe using messages are an important part of reducing drug-related harm, but people working with IDUs must consider the context in which risk behaviours occur and efforts to reduce said behaviours must include attempts to reduce environmental risk factors at the same time.

## Background

Injecting drug users (IDUs) experience higher rates of death and poorer health than their non-injecting peers. IDUs are between 6 and 20 times more likely to die than their non-heroin-using peers of the same age and gender [1]. Death due to suicide among heroin users occurs at 14 times the rate of matched peers [2]. The major type of heroin-related mortality and morbidity is heroin-related overdose. At the time of this study, the number of deaths attributed to opioid overdose in Victoria had risen from 49 in 1991 to 331 in 2000. In Australia, around a quarter of heroin users report having experienced an overdose in the past 6 months, and over 70% reporting having witnessed an overdose in the previous 12 months [3-5]. The other major cause of mortality and morbidity in IDUs is the transmission of blood-borne viruses (BBVs), most usually HIV and hepatitis C (HCV). The high prevalence of HCV infection, and the increased infective ability of HCV in comparison to HIV, makes sharing of all forms of drug paraphernalia, not simply needles, a high-risk practice [6]. In addition to the risk of overdose and BBV transmission, environmental factors such as the illicit status of heroin, stigmatisation of IDUs and barriers to effective treatment maximise the consequences of risky behaviour. These factors combine to create an environment where death and disability are common occurrences for IDUs and this study seeks to document IDU attitudes towards death and the relationship between these attitudes and health promotion strategies.

Health promotion strategies (such as health education programs) have shown some success in the general population and much of this thinking has influenced the programs implemented with IDUs such as 'safe using messages' aimed at preventing overdose and BBV transmission. However, there is a small, but growing, literature which documents examples of when human desires and preferences mean that health behaviour is prioritised lower than other considerations. This has been seen in regard to the use of condoms [7,8], dietary habits [9,10] and smoking [11,12]. The majority of interventions targeted at overdose have revolved around 'safe using messages'. Typical messages include: 'don't mix your drugs', 'split the dose', 'always use with a friend', 'use where you can be found' and 'watch your tolerance' [13]. Whilst there is abundant literature describing program implementation of safe using messages, there are no evaluative studies of such strategies. The main intervention targeted at reducing BBV transmission has been needle and syringe programs (NSPs) and their associated safe using messages. Such messages include: 'don't share needles', don't reuse needles' and 'don't share other injecting equipment'. Because of the logically combined nature of these interventions, the effectiveness of health promotion messages alone remains untested, but such programs appear to have limited success in reducing some harms compared to others, especially in relation to overdose prevention and HCV transmission. While there have been some investigations around risk behaviour in marginalised groups [e.g. [14,15]], these studies have not investigated the role of attitudes towards death and how indifferent attitudes affect the relevance of health promotion messages. This study sought to understand some potential barriers for IDUs acting on health information, investigating their attitudes towards drug taking and death and how such attitudes may impact upon the effectiveness of safe using messages.

### IDUs' attitudes towards risk

Most IDUs report never or rarely worrying about overdose or BBV transmission (excluding HIV/AIDS) [14,16]. Though not well studied, prior studies have also shown that engaging in high risk behaviours does not necessarily mean that someone has a reduced fear of death [17]. In some instances, individuals will act to minimise risk that is an unavoidable part of their environment, while still engaging in risky behaviours. For instance, crashes and death in serious recreational cyclists, a pursuit that involves regular brushes with death, are viewed as inevitable or unavoidable and are seen as 'occupational hazards' [18]. Although the high level of danger is constant, cyclists are not actually 'death cheaters'. Rather, "due to the unavoidably risk-laden nature of the activity, the subculture of cycling has incorporated the dangers of riding in ways that inextricably linked them to the very enactment of that life, the bike life" [[18]: 169]. Many risk takers, (e.g. parachutists and cyclists) often carefully try to reduce the risk as far as possible, but in some cases, such as cyclists, environmental factors such as the dominance of cars on the road, mean that the hazards they are exposed to are substantially increased and beyond their control. Similar attitudes have been theorised for soldiers in conflict situations, particularly those from lower class backgrounds [19,20]. In their case, it has been suggested that indifference towards death is socially constructed through the dual masculinised roles of both "a man" who carries arms, trained to kill and to cope with the death of a close friend, or a "real man" who takes care of, and provides for, his family [19]. Both roles ultimately view death as an occupational hazard, though, like cyclists, they are not indifferent to their fates and take all reasonable precautions.

### The Social Risk Environment

The perception of risk is highly contextual and it is worth considering that risk can not only be enjoyed or avoided, it can also be ignored. For example, Plumridge and Chetwynd [21] also observed that, for some of their sample, risk was not denied or overridden, but acknowledged. This can also be affected by the individual's self constructed identity and the social environment they inhabit. They noted that IDUs can inhabit "a social world in which there was very little sense in which anything other than drug taking provided a raison d'etre" [21].

People are often driven by ambivalent and confused motives, such as a desire to achieve relief from pain or to escape an unbearable situation [15,21,22]. While most individuals will reject the role of social/structural determinants on their behaviour, preferring individualistic explanations that affirm self-efficacy [21,23], research consistently identifies how the social and structural environment we inhabit influences our behaviour, particularly in relation to drugs [23,24]. Specifically, the relationship between poverty, its consequent marginalisation and risky drug-taking behaviour is well documented [25,26].

This relationship is even stronger when urban deprivation is found in combination with vulnerability and trauma [27,28]. Deprived urban settings are often violent and depressed contexts in which hope of attaining socially ordained norms such as career, wealth and status are only attained by the token few. In such settings, risk and death can be less unattractive than a desire to relieve existential pain, or escape a sense of hopelessness [29]. Importantly, such drug use and attitudes towards risk reflect the reality that drug use can be functional, pleasurable, problematic and dangerous at the same time [26]. Within such a personal and social milieu, reduction of harm may not be prioritised.

## Methods

Sixty heroin users were interviewed over a six week period in April/May 2000 at two needle and syringe programme (NSP) sites in Geelong, Australia. The sample was a convenience sample and interview subjects were recruited using contact cards handed out by outreach workers, NSP workers and ambulance paramedics attending overdose events. The recruitment card informed potential participants that interviews were about risk and heroin use. To be eligible for the study, subjects had to have used heroin in the previous month. Interviews were conducted in interview rooms provided by Barwon Health Drug and Alcohol Services. Ethical clearance was granted by both Deakin University Human Research Ethics Committee and Barwon Health Research Ethics Committee. Access to counselling was provided if required as well as referral for other support services. No interviewees requested counselling, although one interviewee was referred to the local psychiatric service following a suicide attempt. Subjects were reimbursed $20 per interview.

Qualitative, semi-structured interviews were used and interviewees were encouraged to talk freely of their experiences and opinions. General discussion topics of interest were listed on a checklist to ensure all interviewees views were sought on each topic. Discussions were not structured in any particular order and topics were ticked off as mentioned in the normal course of the more general discussion. All interviews were recorded and transcribed verbatim. Interviews took between 20 and 95 minutes and subjects were required to use a pseudonym to ensure anonymity. Participants were asked about overdose patterns, blood-borne virus behaviour, suicidality and attitudes towards death [29-31]. They were specifically asked about their risk behaviours, attitudes toward death, whether they had ever attempted suicide and were engaged in subsequent conversation regarding details on each topic such as triggering events and other contextual details. While the study also looked at suicidal thoughts and behaviour, these findings are presented elsewhere [29]. All questions were read out during the interview.

### Setting

Geelong is a city of approximately 205,000 people with a growth rate of 1.1% per annum. Located 70 kilometres from Melbourne, it is both a regional centre and a suburb of Melbourne. Geelong is traditionally and industrial and port town, but has seen massive decline since the 1970s and now has few large manufacturers remaining. This working class basis and subsequent decline in employment has seen a raft of social problems over the past 3 decades, with alcohol, drugs and drug-related violence featuring prominently on the social landscape. A number of traditional working-class suburbs have become dominated by social housing, unemployment and social security dependence. Most interviewees reported currently living in these suburbs, although it is unclear how long they have lived there and over half reported unstable housing.

### Analysis

Statistical analysis was conducted with SPSS and qualitative data was analysed using NVivo. The narratives in this article result from thematic categorisation. Thematic analysis is an inductive design where, rather than approach a problem with a theory already in place, the researcher identifies and explores themes which arise during analysis of the data [32]. In this analysis, once a theme became evident, all transcripts were reanalysed for appearances of the theme. Categorisation was not exclusive and some narratives appeared in many themes. Categories are added to reflect as many of the nuances in the data as possible, rather than reducing the data to a few numerical codes [33]. All the data relevant to each category were identified and examined using a process called constant comparison, in which each item is checked or compared with the rest of the data to establish analytical categories. For the sake of transparency, results reported are enumerated [34]. Where available, narratives which present opposing viewpoints will also be presented [35].

### Limitations

The aim of this article is not to present an exhaustive analysis of this data, but to offer some insights on indifference and injecting drug use using this qualitative material. To this end, and considering the relatively small sample size, the findings presented here are not generalisable. In addition to this, the thematic coding undertaken was conducted by a single researcher and may therefore be open to interpretation. The study is also limited in terms of its limited geographical range and the possibility that different localities will carry different cultures around risk, although this was not evident in the available comparisons such as rates of overdose and needle-sharing. The study also lacked a stated sampling frame, simply using a convenience sample of people who attended NSPs. It is possible that more a more structured sampling frame, combined with a larger sample, may have identified differences within sub-groups of IDUs in relation to risk behaviour and attitudes towards death.

Finally, the study ultimately relied on self report. While self report has been found reliable in relation to behaviours which are able to be measured though other means [36], it is unwise to assume that self report will be reliable for all aspects of a person's behaviour. In particular, when talking about death and risk, it is possible that a number of interviewees displayed some bravado or other reasons for reporting in a socially constructed manner. Previous research has identified that there are many factors which might affect the way in which interviewees wish to present themselves. Interviews are firstly a socially interactive enterprise. "Evidence of such reflexive organisation of the self can be seen in individuals' sensitivity to social circumstance and sanction in relation to their identities' [21]. Motivations can include the preservation of personal self constructions such as heroic individualism, responsibility, maturity, courage or weakness which ultimately reflect their sense of moral worth. Ultimately, interview accounts are constructed by actors interested in achieving certain social effects in their story-making concerning identity, reflexive biography and, for the purpose of this study, agency concerning risk management and attitudes towards death [37,38]. On the other hand, it is worth considering that most self report data has aligned with other research evidence [21].

## Results

Most of the interviewees (n = 36) were male (see Table 1). The average age of interviewees was 28.1 years old (range 15–51 years). All interviewees had used heroin within the past week and most reported that their main 'drug of choice' was heroin. Over half (53%) of interviewees were not currently in treatment and 30% were in methadone maintenance treatment (MMT).

**Table 1:** Summary Statistics

Mean Age (range) yrs	28.1 (15–51)
Median Age yrs	26.0
N Male (%)	36 (60%)
Education, N (%)	
- year 10 or less N (%)	33 (55%)
- commenced university	3 (5%)
Employment, N (%)	
-unemployed	36 (60%)
-pension/disability support	12 (20%)
-part-time employed	9 (15%)
-students	3 (5%)
Accommodation, N (%)	
-homeless	22 (36%)
-rental	19 (32%)
Drug of choice, N (%)	
- Heroin	55 (92%)
- Amphetamines	3 (5%)
- Cannabis	2 (3%)
Heroin Use Duration	
-Mean (SD)	7.4 yrs (7.37)
-Range	1–30 years
Treatment, N (%)	
-not in treatment	32 (54%)
-methadone maintenance	18 (30%)
-counselling	6 (10%)
Overdose:	
- At least once	35 (58%)
- mean (SD)	4 (SD = 3.79)
-median	3
-range	1–15
Blood borne viruses:	
- HIV tested	54 (90%)
-HCV tested	52 (87%)
- HIV +ve	0
- HCV +ve	32 (54%)
- HBV +ve	2 (3%)

### Overdose experiences and prevention

Over half of the 60 interviewees (n = 35, 58%) report having previously overdosed, with an average of 4 (SD = 3.79) previous overdoses. Thirty two percent (n = 19) of interviewees reported doing nothing to prevent overdose.

### BBV experience and risk behaviours

Interviewee behaviour regarding testing and risk behaviour around BBVs can be seen as possible indicators of the behaviours are able to engage in if they are not ambivalent towards their own fate, as well as a measure of the harm they have already experienced. A substantial proportion of interviewees were unaware of their HIV or HCV serostatus (13% and 10% respectively). Over half (54%, n = 32) were HCV positive and none were HIV positive. Around one in five of the interviewees in this study self-reported both ever borrowing someone else's needle (18%) and lending their needle to someone else (22%).

### Attitudes towards Death

Two questions about death were asked. The first question asked the participant whether or not they ever talked about death with their peers. Most (84%, n = 50) reported that they never talked about death, although 3% (n = 2) reported that they often discussed death as a possible consequence of their heroin use. Interviewees were also asked how they felt about death and whether they were afraid of dying. The vast majority (82%, n = 49) stated that they were never afraid of dying, 12% (n = 7) said that they were afraid of dying from some causes other than heroin use (i.e. car accident) and 3% (n = 2) of the interviewees reported that they were often afraid of dying. Narrative responses showed that almost half of the interviewees (n = 28) were either indifferent or fatalistic about death.

*Wayne, 51 yrs, Well, I surely don't want to die, but it doesn't make me not want to use. If it did I wouldn't use any more, because I've dropped a few times. It hasn't frightened me off enough. I know if I die, I'll just go to sleep any way, I just don't wake up*.

Wayne's narrative provides an example where overdose death is perceived to be a comparatively pleasant experience. This attitude can be seen in its extreme form in the following narrative.

*Casey, 15 yrs, I reckon that was the best feeling, overdosing. The best feeling ever. The first time I ever felt so stoned. It was just the best feeling ever. There was a time when I was apparently dead. It was grouse, I felt like a was asleep and I was just going through this full trippyness. It was the best feeling*.

Casey's narrative holds a number of insights into both the motivation for risky heroin use, but also could be an example of the bravado expressed by a young person discussing a frightening experience. In the context of a research interview, and the complexities of such a social interaction, it is probable that both elements are at play. Ten interviewees also reported indifference towards both life and death.

*Peter, 28 yrs, ...sometimes it gets too much. You're broke all the time. You haven't got a roof over your head or you haven't got money for food. You just get sick of the lifestyle. It's a real bugger because it's something you love but you get discriminated against. You know, the way people treat you, even your family. It [heroin overdose] would be a good way to go, better than cancer*.

Peter's narrative points to many factors related to poverty and urban deprivation, in addition to dependence on heroin. Peter is also clear that the consequences he identifies are primarily social or societal in their origin, including a lack of accommodation, the lack of money or food, and more general discrimination, which are also mostly out of the control of the individual IDU. Such narratives suggest that poverty and urban deprivation play a role in IDUs attitude towards life, death and risk.

Another major theme to arise from the narratives (n = 8) was that death was an occupational hazard of heroin use.

*Frank, 24 yrs, I think that people who use accept that as one of the risks. You just cop it on the chin*.

*Joe, 31 yrs, nearly every time, I know its Russian roulette. Sometimes pills. Also some speed, usually hammer first, then speed. Dropping is really an occupational hazard. When your number's up, your number's up. Why worry about it. It's just as likely that you'll have a good whack and then walk across the road and get hit by a truck*.

Finally, not all interviewees exhibited the above-described attitudes towards death and three interviewees reported that they were not indifferent towards death and did their utmost to avoid death.

*Bruce, 23 yrs, I mean, you talk about friends that have died and that, but I don't really have any sympathy for them. It sounds a bit harsh, but like I say, I've had a lot of friends that have died from one way or the other, you know, but if it's through the choices they made then that's their own business, you know what I mean. I don't want my daughter to know her whole life that her dad died a junkie*.

*David, 35 yrs, it is out of control in one sense but I don't break into houses or anything like that. The only control I have is to throw myself into an area where it's impossible to get heroin. The best I can do is one day without. There use an element of control I suppose, but it's not enough to break free. I don't want to die. Either that or fail heroicly*.

### Worst Consequences

Interviewees were also asked what they believed would be the worst consequence of experiencing an overdose. The interviewees were then read a list of four possible alternatives and asked to nominate one (death, brain damage, police involvement or being woken up). The order of consequences was randomly altered. Interviewees were also able to identify other consequences from which three more responses were identified (nothing, all and wasted money).

Whilst thirteen interviewees reported that death was the worst consequence of overdose, the majority (58%, n = 35) of interviewees identified brain damage as the worst possible consequence of an overdose. Other responses included 'Being woken up' (n = 5, 8.3%) and 'Police Involvement' (n = 3, 5.0%). Whilst this finding is similar to responses from non-IDU populations [39,40], it does demonstrate that the majority of these interviewees clearly identified that there was something worse than death. For example:

*Lisa, 25 yrs, I knew a guy who overdosed and ended up with brain damage and he ended up brain dead and they turned the machines off. That was pretty sad really. With my partner, I think about it: is it bad for him to be here brain-dead or with brain damage. I think I'd prefer them to die than have brain damage, but then it's the people that they leave behind. I think a lot of families go through a lot of shit. I mean, it's hard to say. Here I am saying "these families go through a lot of shit", but then I'll go and risk killing myself. For me in an overdose, I'd prefer to die, than have fucking brain damage*.

The next most common response was 'being woken up'.

*Damian, 29 yrs, Coming back with a fucking Narcan headache. That's worse than anything I've ever had. I'd definitely rather be dead than brain damaged. It's part of the game isn't it, guaranteed, you're born to die*.

*Debbie, 22 yrs, for me it was just waking up, that was the pits. For the person overdosing the worst consequence is waking up straight. If you've got people with you, you shouldn't get brain damage. All they're concerned about is the drugs and getting drugs and being stoned*.

## Discussion

The data presented above highlights that many interviewees did not see the possibility of dying as a reason to reduce risk behaviours. Most experienced the consequences of their risk behaviour regularly, yet few reported engaging in safe using practices. Despite the fact that death is a common occurrence in this group of people and they engage in a behaviour that carries a risk of death every day, most tend to repress their fear of death, treating the likelihood of their death with either ambivalence or indifference. It was apparent that when the effect desired from drug use is on the verge of overdose/death, safe using messages are unlikely to be of sufficient priority. For example, telling an IDU to 'taste' their heroin prior to using the whole amount makes little sense to someone attempting to gain the maximum effect from the heroin they possess. These findings raise questions about the conclusions arising from the existing literature which focuses on changing individual behaviour and suggests support for interventions based on reducing environmental risk [41].

### IDUs relationship with death

The major finding of the study is the high level of indifference and fatalism displayed by many of the interviewees towards their own death and the way in which social and environmental factors such as poverty and marginalisation form the background for this indifference. The narratives support the observations of previous research that many of the interviewees were driven by ambivalent and confused motives [22]. In particular, it was observed that for some IDUs, their death is an event which is viewed with some dispassion and taking measures to try to avoid the death can appear to be the equivalent of 'avoiding the unavoidable'.

The narratives presented also lend weight to the proposal that indifference towards death may turn out to be a rationally based response to "social isolation, meaninglessness and anomie, so characteristic of social life in the 20th century" [[42]: 715]. They point to the reality that in the lived experience of these IDUs where "health may be accorded a relatively low priority by individuals suffering psychological difficulties or social deprivation" [[43]: 223]. Indeed, it is implicit in these narratives that many of the interviewees inhabited a social sphere where there was very little in their lives that supplied meaning apart from substance use, similar to that proposed by Plumridge and Chetwynd [23].

Indifference might also be seen as a matter-of-fact response to the very high death rate amongst heroin users, but can also be viewed as fatalistic. Accepting risk as an 'occupational hazard' may tacitly be denying any sense of agency towards risk behaviour and may result in IDUs not engaging in risk avoidance behaviours. However, the idea of an occupational hazard is common amongst other groups within Western society that engage in high levels of risk behaviour. As seen in Albert's investigation of risk and injury in serious recreational cyclists [18], the concept of occupational hazard is employed widely to deal with situations which, on-the-whole, have little to do with the individual's behaviour and are more related to societal norms surrounding automobile use. The concept of death as an occupational hazard attitude is also reflected in some discourses of soldiers in wartime settings [19,20,44]. However, the literature on attitudes towards death in wartime soldiers emphasises more strongly the conflict-laden nature of such attitudes, particularly in relation to dual roles of masculine provider and citizen. The narratives from interviewees in this study also referred to conflicting elements of their life, most particularly the need to feed their habit while staying alive. From this sample, it was difficult to draw any inferences about gender roles in this regard, although a confrontational attitude towards death was more apparent in men. However, the clearest parallel was the maintenance of the self image in a hostile environment, where heroin use was viewed as a personal behaviour that was made life threatening because of the drug's legal status.

In the context of drug prohibition inhabited by these interviewees, heroin use has an 'unavoidably risk-laden nature' which leaves the IDU no other option than to reasonably accept death as an occupational hazard of heroin use [15,41]. Thus, it appears somewhat incongruous to suggest that IDUs should use in a safe environment when no such environments exist and illustrates the logic of environmental interventions such as safe injecting facilities. On the other hand, it is also evident that some IDU are neither socially marginalised nor will they choose to use such safe environments, and that the intersection between risk, pleasure, escape and indifference means that reducing harm is not a priority.

The narratives of interviewees have also suggested how such societal factors can impact on a person's indifferent state and have illustrated the link between the mental state of the individual, the high cost of heroin and, by association, current drug policy. Similarly, the recognition by the individual that they are realistically unable, to change situations for themselves, ultimately leads to indifferent and fatalistic attitudes towards their own well-being. When individuals are dislodged from the social fabric of society, or have their aspirations consistently thwarted, they are more likely to hold indifferent attitudes towards their death [45]. This was evident in those interviewees who reported being ambivalent towards life as much as being indifferent towards death. Such attitudes have often been documented in socially and economically deprived urban areas [24,25]. It was also reflected in some responses which relayed a sense of bravado towards death and risk, although the process through which individuals developed such responses and the implications that such attitudes have for understanding their attitudes towards death are most probably derived from a combination of an individual interpreting past events in a way which allows maintenance of self image, as well as the telling of their story which reflects the same goal. Far beyond simple epidemiological correlations, ethnographic work has demonstrated how complex economic, social and cultural factors interact to create situations where drugs become a central part [15]. In such a social environment, "people who are using don't care". These findings suggest that beyond investigating and treating drug use, "poverty and deprivation warrant intervention in their own right" [26].

## Conclusion

Most of the interviewees in this study were indifferent towards heroin-related death. Whilst interviewees were well aware of the possible consequences of their actions, these consequences were not as important as achieving their desired state of mind. Despite the fact that death is a common occurrence in this group of people and they engage in hazardous behaviour on a daily basis that carries a risk of death, most treat the likelihood of their death with either indifference or resignation. When marginalised groups such as IDUs experience the 'existential angst' observed in some of the narratives presented above, messages of harm reduction and health promotion may be of little relevance.

These findings illustrate that it may be more important to address the reasons behind this indifference than to attempt to change behaviour. In the current drug policy context of prohibition, discourses surrounding the rational choice of IDUs to reduce the risk associated with their drug use are sometimes simplistic and unrealistic. In reality, many IDUs do not have a full range of choices of how to reduce the risks associated with their drug use and the discourses of choice espoused within safe using messages may ultimately fail to serve the drug user and the wider community, encouraging 'victim blaming' thereby further entrenching the marginalisation and fatalism of IDU populations.

## Competing interests

The author declares that they have no competing interests.

## Authors' contributions

PGM conducted all elements of this study
